# Improvement of Corrosion Resistance of TiO_2_ Layers in Strong Acidic Solutions by Anodizing and Thermal Oxidation Treatment

**DOI:** 10.3390/ma14051188

**Published:** 2021-03-03

**Authors:** Badar Minhas, Sahib Dino, Yu Zuo, Hongchang Qian, Xuhui Zhao

**Affiliations:** 1 School of Material Science and Engineering, Beijing University of Chemical Technology, Beijing 100029, China; badar.minhas@outlook.com (B.M.); Sahib.gul871@hotmail.com (S.D.); 2 Institute for Advanced Materials and Technology, University of Science & Technology Beijing, Beijing 100083, China; qianhc@ustb.edu.cn; 3 Beijing Key Laboratory of Electrochemical Process and Technology for Materials, Beijing University of Chemical Technology, Beijing 100029, China

**Keywords:** Ti, anodized film, thermal oxidation, harsh environments, corrosion resistance

## Abstract

By anodization and thermal oxidation at 600 °C, an oxide layer on Ti with excellent corrosion resistance in strong acid solutions was prepared. The structural properties of TiO_2_ films before and after thermal oxidation were investigated with methods of Scanning electron microscope (SEM), X-ray photoelectron spectroscopy (XPS) and X-ray diffraction (XRD). The electrochemical characterization was recorded via electrochemical impedance spectroscopy, potentiodynamic polarization and Mott–Schottky methods. XRD results show that a duplex rutile/anatase structure formed after oxidation, and the amount of anatase phase increased as the treatment time was prolonged from 3 to 9 h. XPS analysis indicates that as the thermal oxidation time increased, more Ti vacancies were present in the titanium oxide films, with decreased donor concentration. Longer thermal oxidation promoted the formation of hydroxides of titanium on the surface, which is helpful to improve the passive ability of the film. The anodized and thermally oxidized Ti samples showed relatively high corrosion resistance in 4 M HCl and 4 M H_2_SO_4_ solutions at 100 ± 5 °C. The passive current density values of the thermally oxidized samples were five orders of magnitude under the testing condition compared with that of the anodized sample. With the oxidation time prolonged, the passive current density decreased further to some extent.

## 1. Introduction 

Due to their inherited unique relationship of sound biocompatibility, excellent chemical stability and high specific strength, titanium and titanium alloys exhibit a great variety of applications covering from aerospace and biomedical to nuclear waste. However, a major barrier for applications of titanium is its poor tribological characteristic, particularly when high wear resistance is recommended. Furthermore, the formed oxide films on titanium are sensitive to damage not only by halide anions but also in other media, such as sulfate solutions [1,2,3]. To overcome these challenges, various surface modification methods have been applied to titanium and titanium alloys. It has been reported that different surface modifications of titanium often show better corrosion resistance. For instance, Liu et al. reported that the composite film TiC-Ti_5_Si_3_ fabricated on substrate Ti6Al4V shows corrosion current density one order lower in magnitude than the untreated Ti alloy in simulated artificial sea water [4]. It is important to understand that for the treated material structural defects presented frequently (e.g., micropores and microcracks) in the coating, these structural defects provide the path by which the active species diffuse and damage the substrate. Hence, new methods for surface modification and new coatings are required to further improve both the mechanical and chemical stability of the oxide films [5,6].

Recently, nanostructured titanium oxide with dual phases (anatase and rutile) has attracted widespread attention to fabricate a structural coating used under harsh conditions due to its low density, excellent oxidation resistance and adequate strength to weight ratio. In the attempts to achieve nanostructured titanium oxide with dual phases, numerous surface modification methods, such as thermal spraying, electrochemical anodization and thermal oxidation, have been reported, while each of the methods exhibits its own limitations and values [7,8,9]. Thermal spraying is a powerful technique in yielding thick titanium oxide coatings, but it is difficult to apply to substrates with intricate shapes. Anodization is an electrochemical technique that has been effectively demonstrated for the surface treatment of titanium and its alloys. Anodization is defined by the rate of formation and dissolution of oxide film specified by the selection of electrolyte and its temperature. By changing the parameters of the anodization process, titanium oxide films with different phases can be formed on Ti substrates [10,11]. In the case of sulfuric acid, the oxide formation rate is much more dominant than its dissolution rate. Through anodization, the formed crystalline oxide is denser and thicker than the native oxide, and the properties of the oxide, such as thickness, morphology, crystallinity, color and porosity, may be controlled by the operation specifications. Sul et al. reported that a reduction in the current density appeared by altering the temperature and concentration of electrolyte [12]. Some of the advantages observed by applying anodizing processes include reduced cost (no necessity of gas shielding or costly vacuum conditions); simple and easy operation variables to fabricate the required coatings; ecological favorability, since there is no harmful emission involved in the operation; good adhesion; and easy incorporation with different surface treatments, such as thermal oxidation [13,14].

Increased corrosion resistance is obtained for anodized titanium by thermal oxidation. Thermal oxidation has been found to be an attractive method which allows the expansion of the anodized film to establish a dense crystalline film dependent on the applied temperature. Anodic films subjected to annealing at 550–600 °C form dual phase structures, which show efficient adhesion and higher corrosion resistance than amorphous or any single phase TiO_2_ [13,15]. However, high temperatures beyond a certain limit and prolonged heating will minimize the functional properties. Prolonged annealing at 800 °C leads to massive building of the oxide film, which ultimately spalls off from the surface due to the difference in the thermal expansion coefficient and lattice mismatch between the oxide and Ti substrate. Hence, it is essential to modify the treatment time and temperature so as to produce thick, homogenous and adherent film, preferably with a dual phase structure [15].

It has been found that the anodization and thermal oxidation of titanium and Ti alloys improve the resistance against degradation. For example, Cimenoglu and Guleryuz [3] reported that commercially pure Ti subjected to thermal oxidation at 600 °C for 60 h showed good inhibition efficiency in hydrochloric acid solution. The result showed that the corrosion resistance of the oxide layer was improved by minimizing the anodic dissolution as compared with untreated Ti. John et al. [9] discovered that Ti6Al4V subjected to anodization followed by thermal oxidation showed superior corrosion resistance to the samples treated separately with anodization or thermal oxidation. This may be because anodization was effective in producing anatase/amorphous TiO_2_, while thermal oxidation efficiently converted the oxides into dense dual anatase/rutile structures, exhibiting improved degradation resistance. The above methods were employed mostly to modify Ti and its alloys for the improvement of biomedical applications, and no organized study was found on Ti and its alloys at high temperatures in strong acid environments. Our recent study [16] found that anodized TiO_2_ films followed by heat treatment at 600 °C built protective layers that showed an anatase/rutile dual-phase structure and excellent corrosion resistance to strong acidic solutions at high temperatures. Based on previous work, in the present study, the hybrid treatment conditions, particularly the heat treatment time, are further investigated to obtain the best corrosion resistance. The corrosion resistance of the obtained oxide films on Ti are studied in 4 M H_2_SO_4_ and 4 M HCl solutions at 100 °C, using conventional electrochemical techniques containing potentiodynamic polarization, electrochemical impedance spectroscopy (EIS) and Mott–Schottky analysis.

## 2. Experimental Methods

### 2.1. Substrate Preparation 

The Ti substrate samples were formed into 30 × 15 × 2 mm by cutting from a commercially pure Ti plate. The nominal composition in wt.% was C: 0.19; H: 0.15; N: 0.017; Fe: 0.20; O: 0.14 and balance Ti. Before electrochemical anodization, the samples were mechanically polished by using silicon carbide abrasive papers down to 1500 grade. After that, the polished Ti samples were washed with ethanol and dried at room temperature. The samples were pickled for 25 s in a pickling solution (HF:HNO_3_ = 1:3). An electrochemical cell was used to perform anodization operation, where the anode and cathode were the Ti sample and a graphite electrode, respectively. The anodization was executed with nonstop stirring in 1 M H_2_SO_4_ solution at 45 °C for 5 h (15 V + 60 V), followed by immediate cleaning after completion with distilled water. After electrochemical anodization, thermal oxidation was carried out in muffle furnace (Model: KJ-M1400-8LZ, Kejia Muffle Furnace, Zhengzhou, China) at 600 °C for 3, 6 and 9 h, respectively, then the samples were cooled inside the furnace to room temperature. As reported elsewhere [9], the appropriate selection of parameters alters the phase structure of titanium oxide into rutile or anatase and has a significant influence on its ability to withstand harsh environments. So, using the above hybrid treatment (anodization + thermal oxidation at 600 °C) with respect to different durations of time, composite oxide films were obtained and the relations between the corrosion resistance and the structural and morphological parameters were investigated. The above-mentioned operation conditions are listed in Table 1.

### 2.2. Composition and Microstructure Characterization 

The identification of different phases in the prepared films before and after thermal oxidation was studied by an X-ray diffractometer armed with Cu kα irradiation. The spectra were obtained from 5°to 90° in 2-theta range. The morphological and compositional analysis of the films after anodization and then further after thermal oxidation were studied by a scanning electron microscope (SEM SU8200, Hitachi, Japan) armed with X-ray energy dispersive spectroscopy (EDS). X-ray photoelectron spectroscopy (XPS) was measured by a spectrometer (Kratos AXIS Ultra ESCA system, San Diego, CA, USA) using Al kα with 20 eV of pass energy at 1486.71 eV.

### 2.3. Electrochemical Measurements

A basic electrochemical cell consists of a three-electrode system used for electrochemical measurements, with the treated substrate (Ti) as the working electrode, Pt as the counter electrode and a saturated calomel electrode (SCE, Xian Yima optoelec Co, Ltd, Xian, China) as the reference electrode. All electrochemical measurements were executed at 100 ± 5 °C in 4 M H_2_SO_4_ and 4 M HCl solutions, using the EG&G model 273A potentiostat (Princeton Applied Research, Princeton, NJ, USA). The potentiodynamic measurements were recorded against SCE at a scan rate of 0.66 mV/s from −1 to 3 V. EIS measurements were performed with a perturbation of 10 mV at open circuit potential, with frequency ranging from 100 to 10 mHz. The EIS experimental data were fitted by using ZSMIPWIN software (Version 3.22, 2010, Princeton Applied Research, Princeton, NJ, USA) to determine an appropriate equivalent electric circuit (EEC). A Mott–Schottky test was conducted against SCE with a perturbation of 10 mV in a range of 0.3 to 2 V with scan speed of 50 mV at 1 kHz frequency, to measure the donor density and the flat band potential. The samples were immersed in the solutions for one hour before each electrochemical measurement. At least three measurements were performed for each electrochemical observation.

## 3. Results and Discussion

**Figure 1:**
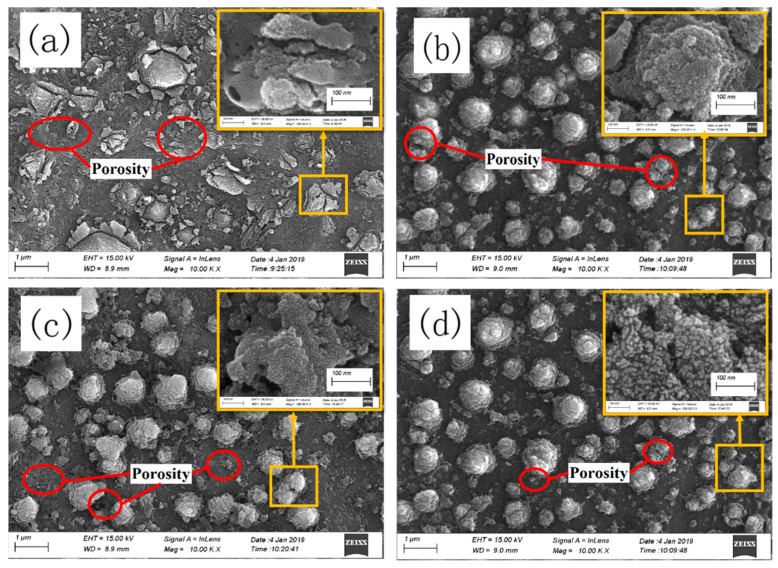
SEM view of the oxide surface for groups (**a**) X, (**b**) Y_1_, (**c**) Y_2_ and (**d**) Y_3_.

Figure 1 illustrates the surface morphology of groups X, Y_1_, Y_2_ and Y_3_, respectively (Table 1). Group X showed porous and irregular TiO_2_ particles with uneven distribution at the whole surface. Groups Y_1_, Y_2_ and Y_3_ showed very similar morphology. Moreover, from group Y_1_ to Y_3_, the amount of porous structure reduced without any change in the morphology, which implies that the grains of the film had adequate thermal stability for a prolonged time period at high temperatures. The magnified view of Figure 1a–d shows a very clear perspective of the film structure; the layers seem to be irregular in shape and size, indicating that a rough surface was formed with a high specific surface area [17,18].

**Figure 2:**
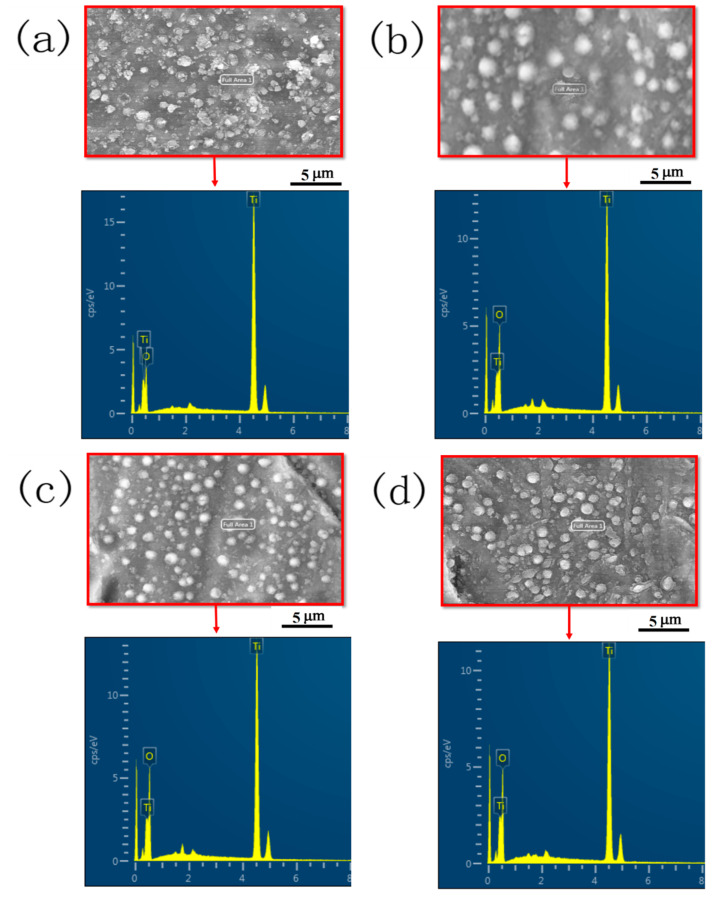
EDS mapping of groups (**a**) X, (**b**) Y_1_, (**c**) Y_2_ and (**d**) Y_3_.

Figure 2 represents the EDS results for the sample surface of groups X, Y_1_, Y_2_ and Y_3_, respectively. As seen from Figure 2a–d, the prepared films are mainly made up of titanium and oxygen. The EDS spectrum of group X shows that Ti peaks are much higher than those of groups Y_1_, Y_2_ and Y_3_. Groups Y_1_, Y_2_ and Y_3_ represent enrichment in oxygen with respect to the thermal oxidation time; the maximum enrichment was seen in group Y_3_. As reported elsewhere [19], the increment in the TiO_2_ particles may be due to the existence of Ti vacancies in the film. According to Vennekamp et al. [20], the growth mode for the formation of TiO_2_ is controlled by the movement of ionic species. If morphologically uneven growth occurs, then Ti ions are more mobile; once oxygen ions are mobile, stable growth is observed.

**Figure 3:**
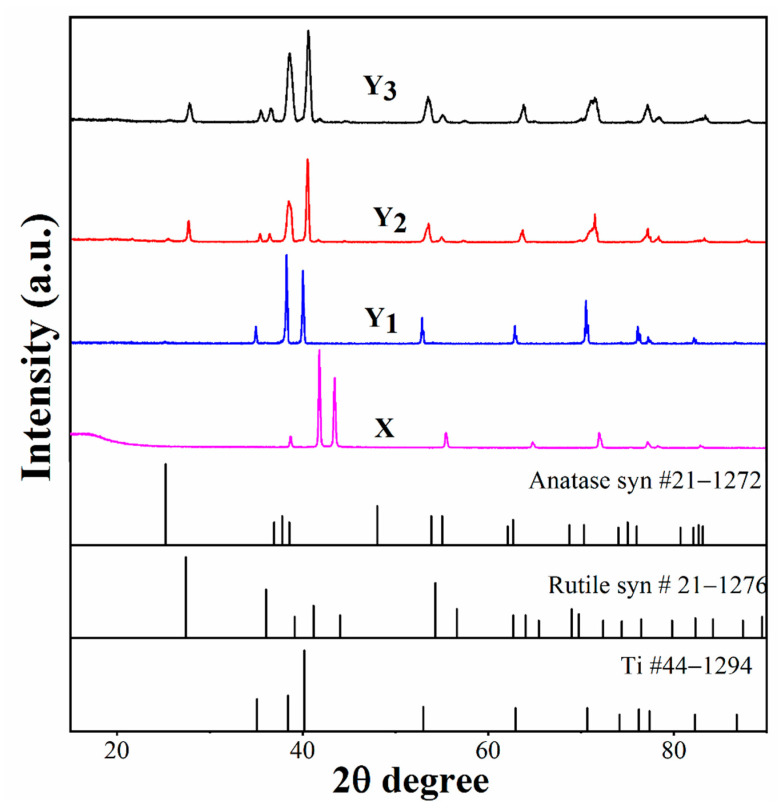
XRD spectra of prepared groups X, Y_1_, Y_2_ and Y_3_.

Typical X-ray diffraction spectra obtained from all groups and the comparison with the standard powder diffraction patterns for TiO_2_ (anatase or rutile) are shown in Figure 3. For group X, the sharp diffraction peaks exhibited nonstoichiometric and amorphous TiO_2_ along with few peaks of α-Ti, which implies that an uneven thin film was produced. The XRD spectrum of group Y_1_ was very similar to those of groups Y_2_ and Y_3_, except for the broadening of peaks. Moreover, some new diffraction peaks were also seen in groups Y_2_ and Y_3_, suggesting that the presence of different phases increased with a decrease in crystalline size [9]. It was reported that the annealing of TiO_2_ at 580–600 °C altered the structure into two polymorphic forms of TiO_2_ (anatase and rutile). In groups Y_1_, Y_2_ and Y_3_, rutile peaks were noticed at 2θ of 36.0 (101), 41.2 (111), 54.3 (211), 62.8 (002) and 70.0 (112), while the new peaks found at 2θ of 27.4 (110), 75.9 (301) and 82.1 (303) are presented as rutile and anatase phases, respectively. In group Y_1_, the peak that appeared at 2θ of 38.0 (004) represents the anatase phase. Careful examination showed that the XRD spectra of Y_1_, Y_2_ and Y_3_ moved towards lower values of the Bragg angle as compared with the standard powder diffraction pattern, suggesting the interstitial areas being saturated by oxygen. Furthermore, in group Y_3_, the peak intensities of the anatase phase were obviously higher than those in groups Y_1_ and Y_2_, indicating that with longer treating time at the testing temperature, more anatase phase in the oxide film was formed [21]. 

**Figure 4:**
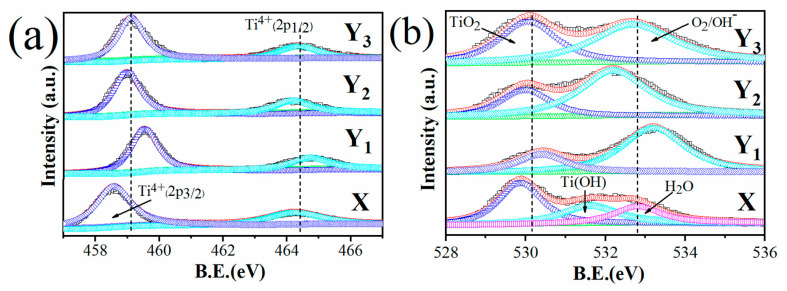
High resolution XPS spectra for groups X, Y_1_, Y_2_ and Y_3_ (**a**) Ti2p (**b**) O1s.

To gain a deeper understanding of the effect of thermal oxidation time at constant temperature, XPS spectra were measured on the surfaces of groups X, Y_1_, Y_2_ and Y_3_. The binding energy values represent the valence state and the density of charge near the atoms. In Figure 4a, the Ti 2p peaks for sample X show the typical binding energy values of Ti^+4^, but the broadened shape on the low energy side reveals the presence of some low value ions of Ti, such as Ti^3+^ and Ti^2+^, similar to previous reports on anodized titanium [14]. After thermal oxidation, the Ti 2p peaks remained in similar shapes, while the major change was observed in O 1s spectra, as presented in Figure 4b. The oxygen spectrum of group X displayed three individual peaks: the main peak located at 529.8 eV is associated with O^2−^ in the oxide, and the two individual peaks clearly resolved in the range of 531–532.7 eV can be attributed to oxygen in the OH group and in H_2_O in the surface layer. This is also consistent with previous authors [2,4]. After thermal oxidation, the peak intensity of O^2−^ in OH and H_2_O obviously increased (Figure 4b). A possible explanation for this is that at high temperatures, oxygen vacancies are generated, and more vacancies are produced if the heating time period is prolonged. In Nishikawa’s study [22], in thermally oxidized Ti film, the atomic ratio of O/Ti decreased with heating, which indicated that the oxygen vacancy in TiO_2_ increased with heating. The generation of Ti^3+^ in TiO_2_ at high temperatures also supports this conclusion. These vacancies can be filled in terms of hydroxyl groups by the dissociation of water that exists in the passive layer, which has been confirmed by the reported literature. Zhang et al. reported that anatase TiO_2_ was exposed to UV light irradiation and a H_2_O environment simultaneously for 60 h, and even after this, the bulk anatase TiO_2_ was preserved due to the formation of an amorphous hydroxylation layer that was very stable and massively hydroxylated over time [23]. He also showed that water plays a significant role in the transformation of titania. The OH peaks that appeared in groups Y_1_, Y_2_ and Y_3_ also confirm this result. According to the TiO_2_-H_2_O Pourbaix diagram, $Ti{\left(OH\right)}_{2}^{2+}$ is the main corrosion product in TiO_2_ films that increases its stability potential in a strong acidic region [24]. The important evidence via XPS analysis shows that thermal oxidation promotes the existence of OH groups on the surface; thus, the TiO_2_ structure is protected by the formation of titania hydroxide. This effectively explained why group Y_3_ had a superior resistance to corrosion.

### 3.1. Potentiodynamic Scan

**Figure 5:**
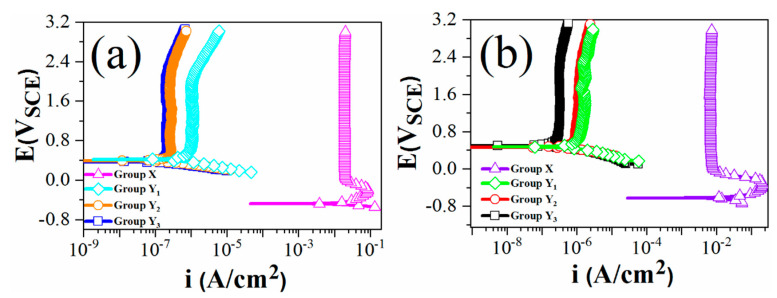
Potentiodynamic polarization curves of groups X, Y_1_, Y_2_ and Y_3_ at 100 ± 5 °C: (**a**) 4 M HCl; (**b**) 4 M H_2_SO_4_.

Figure 5 illustrates the polarization curves of groups X, Y_1_, Y_2_ and Y_3_. The Tafel method was used to determine the E_corr_ and I_corr_ of various samples in 4 M H_2_SO_4_ and 4 M HCl solutions and shown in Table 2. The measured corrosion potential at 100 ± 5 °C for group X in 4 M H_2_SO_4_ and 4 M HCl solutions was about -0.62 and -0.70 V_SCE_, and active-passive behavior was seen. For groups Y_1_, Y_2_ and Y_3_, the corrosion potential increased in both solutions, and spontaneous passivation was achieved. The passive current densities were five orders of magnitude lower than those of group X, showing that the anodizing plus thermal oxidation treatment greatly improved the corrosion resistance of groups Y_1_, Y_2_ and Y_3_. Group Y_3_ showed the lowest passive current density in both solutions, but similar passive current density was also found for group Y_2_ in 4 M HCl. This may be due to the fact that TiCl_3_ was formed in HCl solution, which hindered the dissolution reaction [24,25]. The improvement in corrosion resistance by anodizing-thermal oxidation may be due to the formation of a stable hydroxide film, which acts as a barrier layer. 

### 3.2. Electrochemical Impedance Spectroscopy 

**Figure 6:**
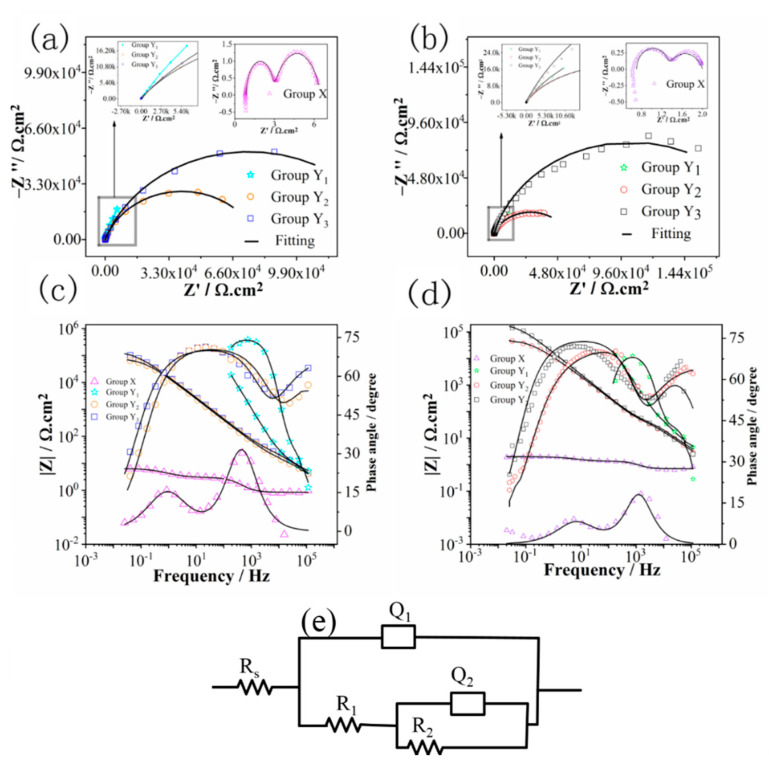
Electrochemical impedance spectroscopy (EIS) plots of groups X, Y_1_, Y_2_ and Y_3_ at 100 ± 5 °C in 4 M HCl and 4 m H_2_SO_4_; (**a**,**b**) Nyquist plots; (**c**,**d**) bode plots; (**e**) equivalent circuit model.

Electrochemical impedance spectroscopy (EIS) is a common method to explore the electrochemical behavior and the reaction kinetics of complicated electrode systems, through which the properties of the electrode materials may be reflected. By careful examination of the EIS data provided by equivalent circuit (EEC) analysis, the entire figure of corrosion procedure can be drawn [4]. Figure 6 displays the EIS plots of groups X, Y_1_, Y_2_ and Y_3_. All the Nyquist plots showed semicircles with different diameters, indicating that all curves were obtained under similar corrosion processes, but possessed different corrosion rates. After thermal oxidation, the diameter of the semicircle increased significantly compared with that of the anodized sample. In 4 M HCl and 4 M H_2_SO_4_ solutions, samples Y_1_ and Y_3_ showed the largest semicircle diameters, respectively, indicating the highest corrosion resistance. Bode plots of groups Y_2_ and Y_3_ are very similar as compared with group Y_1_. According to the bode phase plots of groups Y_2_ and Y_3_ at high and low frequencies, they reflected the capacitive features in the entire frequency range, while group Y_1_ showed capacitive and resistive responses in low and high frequency regions. The corrosion rate of the specimen according to the bode plots at low frequency accelerated in the order Y_3_ < Y_2_ < Y_1_ < X. 

For the electrochemical corrosion evolution of groups X, Y_1_, Y_2_ and Y_3_ by EIS, two time constants were seen in the bode phase plots of all groups; thus, the equivalent electrical circuit (EEC) model (R_s_(Q_1_(R_1_(Q_2_R_2_)))) shown in Figure 6e was used to calculate the parameter values. The choice of the equivalent electrical circuit is dependent on the behavior of the plots, as seen from Figure 6c,d; two time constant were seen, and the fitted data were in good agreement with experimental data throughout the entire frequency range [26]. As reported previously, [4,9] the most appropriate circuit for the analysis of TiO_2_ is (R_s_(Q_1_(R_1_(Q_2_R_2_)))). In the electric circuit (R_s_(Q_1_(R_1_(Q_2_R_2_)))), R_s_ is the solution resistance, Q_1_ is the constant phase element (CPE) for the double layer and R_1_ belongs to resistance at the passive layer via charge transfer at the solution interface, and R_2_ and Q_2_ belong to the resistance and capacitance of the passive layer, respectively. It should be noted that many different justifications have been proposed regarding the circuit in the previous literature. For example, Heakal et al. [27] proposed that the time constant at high frequency related to the capacitance of the double layer and charge transfer resistance may be attributed to the electrochemical process at the solution interface of the passive layer, while the time constant at low frequency is associated with the barrier properties of the passive layer. Some reported data have proposed that the circuit element Q_2_R_2_ indicates the impedance of the passive layer, while Q_1_R_1_ represents the electrochemical response of the passive layer. In the present study, values calculated by equivalent capacitance at high frequencies refer more to the Helmholtz double layer in lieu of the passive layer, because they are generally reported as double layer capacitance values [28].

In Table 3, for all specimens, the passive layer resistance (R_2_) in both solutions was higher than the resistance at the solution interface (R_1_) of the passive layer, indicating that the ionic transport that should rely on the passive layer was predominant, instead of resistance at the solution interface (R_1_) of the passive layer. Based on the analysis of passive layer resistance, group Y_3_ showed significantly higher corrosion resistance than X, Y_1_ and Y_2_ in both solutions. Potucek et al. [29] suggested that the resistance values obtained from EIS data are strongly dependent on the selected solution, while the capacitance (C) is not changed by solution conditions and can yield reliable evidence for the related corrosion properties. The value of capacitance (C_2_) for the inspected groups can be acquired from (Q_2_) work conducted by Hsu and Mansfeld’s using Equation (1) [30]. An approximate estimation for the passive layer thickness (δ) was calculated by using Equation (2)

(1) $${C=Q}^{1/n}R^{\left(1-n\right)/n}$$

(2) $$\delta =\frac{\varepsilon \varepsilon_{0}}{C}$$

In Equations (1) and (2), *R* and *C* show passive layer resistance and capacitance, respectively. ε_0_ (8.85 × 10^−14^ F/cm) represents vacuum permittivity, and ε displays the passive layer’s dielectric constant (ε = 48 for anatase and 110 for rutile). In Figure 3, group X does not show either rutile or anatase peaks, so their values of thickness for the passive layer were not computed here. The ε is taken to be 79 for groups Y_1_, Y_2_ and Y_3_ (the average value of rutile and anatase), due to the anatase or rutile phase [6]. The calculated thickness of the passive layer increased in the order X < Y_1_ < Y_2_ < Y_3_.

### 3.3. Mott–Schottky Analysis

As is well known, the growth and perturbation potential of passive layers mostly rely on the electronic and ionic transport mechanism, and may be measured by the electronic properties of the passive layer. So, it is relevant to examine the electronic behavior of the passive layer to determine the appropriate corrosion process. The Mott–Schottky test is dependent on the experiences of the nonideal semiconductor path followed by the passive layers, and has been demonstrated to be a valid method in studying electronic properties, as defect density can be measured in the passive layer. By Mott–Schottky curves, the connection in (1/C^2^ vs. E) the passive layer may be developed to measure the donor density (N_D_) and flat band potential (E_FB_) [4]

(3) $$\frac{1}{C^{2}}=\frac{2}{\varepsilon \varepsilon_{0}eN_{D}}\left(E-E_{FB}-\frac{KT}{e}\right)$$

Here, e shows the charge of the electron, ε_0_ represents the vacuum permittivity and ε displays the passive layer dielectric constant. In Figure 3, group X does not show either rutile or anatase peaks, so both donor density and flat band potential values were not computed here. The value of the dielectric constant for groups Y_1_, Y_2_ and Y_3_ is considered to be 79. K stands for Boltzmann constant; T and E_FB_ are the temperature of the test solution and flat band potential, respectively. The term kT/e can be ignored, since it is only 32 mV at the required test solution condition. By re-arranging Equation (3), the donor density can be measured and may be obtained by the slope of the best linear fit line as a function of potential of the experimental C^−2^:

(4) $$\mathrm{Slope}=\frac{2}{\varepsilon \varepsilon_{0}eN_{D}}$$

**Figure 7:**
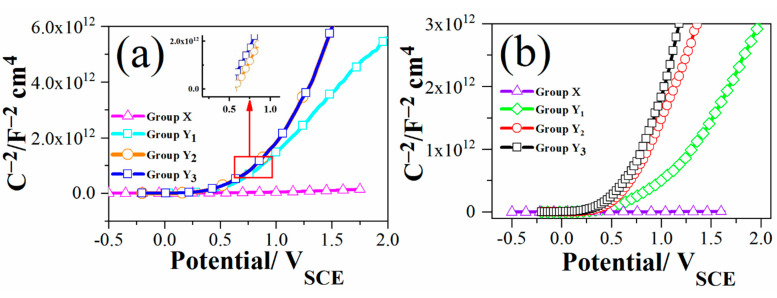
M-S plots of groups X, Y_1_, Y_2_ and Y_3_ at 100 ± 5 °C: (**a**) 4 M HCl; (**b**) 4 M H_2_SO_4_.

The extrapolation of the linear portion to C^−2^ = 0 can be used normally for the measurement of the flat band potential. The measured donor densities of groups Y_1_, Y_2_ and Y_3_ in both test solutions are presented in Table 4. By using this method, the measured donor density indicates the density at the interface of film, whereas the highest concentration of oxygen vacancies and interstitial metal atoms is predicted [4]. From the point defect model (PDM), the whole corrosion process through passive the film is governed by the movement of cation vacancies/or oxygen vacancies, which deduces the resistance to corrosion. So, the main cause is the passive current density that can be determined by the conduction of ions. As reported elsewhere, the greater the concentration density of carriers, the faster the passive film conduction. Consequently, the greater density of a donor will precede higher current density values, which is comparable with the polarization measurement in Figure 5. Normally, the literature N_D_ values rely on the film thickness and electrolyte, and are found to fall in the range 10^18^ to 10^23^ cm^−3^ [31]. The previous results are also acceptable, in agreement with the present results that group Y_3_ has the minimum donor density value. Figure 7 shows that the donor density for group Y_3_ decreased significantly, which may be due to the hydroxide covered TiO_2_ structure. The measured donor density values can be manipulated in both solutions from the highest to the lowest as X > Y_1_ >Y_2_ > Y_3_. The remarkable reduction in the donor density and the prominent improvement in the flat band potential of group Y_3_ imply that the anodization and thermal oxidation improved the protective ability of the films significantly.

In summary, the present work confirms that by anodizing plus thermal oxidation at 600 °C, a protective oxide film was formed on titanium. The film showed good stability in strong acidic solution at a high temperature (100 °C). With the oxidation time prolonged from 3 to 9 h at 600 °C, the corrosion resistance of the oxide showed an increase to a certain degree. The significantly improved corrosion resistance may be attributed to the formation of a rutile/anatase duplex structure, the increased hydroxides on the surface and the decreased donor density in the oxides. 

## 4. Conclusions

By anodization and thermal oxidation at 600 °C, an oxide layer on Ti with excellent corrosion resistance in strong acid solutions was prepared. The effects of oxidation treating time on the film properties were investigated with the following conclusions:

1. XRD results show that a rutile/anatase duplex structure was formed after oxidation at 600 °C, and the amount of anatase phase increased with decreased crystalline size, as the treatment time was prolonged from 3 to 9 h.
2. XPS analysis indicates that as the thermal oxidation time increased, more Ti vacancies were present in the titanium oxide films, with decreased donor concentration. Longer thermal oxidation promotes the formation of hydroxides of titanium on the surface, which is helpful to improve the passive ability of the film.
3. The anodized and thermally oxidized Ti samples showed relatively high corrosion resistance in 4 M HCl and 4 M H_2_SO_4_ solutions at 100 ± 5 °C. The passive current density values of the thermally oxidized samples were five orders of magnitude under the testing condition compared with that of the anodized sample. With the prolonged oxidation time, the passive current density decreased further to some extent.

## Figures and Tables

**Table 1:** Treatment methods and sample labeling.

Group Name	Treatment Condition
X	Anodized
Y_1_	Anodized+ thermal oxidation (3 h) at 600 °C
Y_2_	Anodized+ thermal oxidation (6 h) at 600 °C
Y_3_	Anodized+ thermal oxidation (9 h) at 600 °C

**Table 2:** The E_corr_ and I_corr_ values determined from Tafel slopes.

Samples	E_corr_ (V_SCE_)	I_corr_ (µA/cm^2^)
X (HCl)	−0.70	121 × 10^3^
Y_1_ (HCl)	0.41	0.76
Y_2_ (HCl)	0.38	0.29
Y_3_ (HCl)	0.36	0.26
X (H_2_SO_4_)	−0.62	51 × 10^3^
Y_1_ (H_2_SO_4_)	0.47	1.38
Y_2_ (H_2_SO_4_)	0.46	1.17
Y_3_ (H_2_SO_4_)	0.49	0.10

**Table 3:** Fitting values of EIS circuit.

Samples	4 M HCl				4 M H_2_SO_4_			
	X	Y_1_	Y_2_	Y_3_	X	Y_1_	Y_2_	Y_3_
R_s_ (Ω cm^2^)	0.8	4.6	2.6	3.1	0.7	3.54	2.6	3.3
Q_1_-Y_o_ (Ω s^n^ cm^−2^)	0.002	1.0 × 10^−5^	2.7 × 10^−5^	9.3 × 10^−6^	0.002	1.58 × 10^−5^	1.88 × 10^−5^	6.9 × 10^−6^
Q_1_-n	1	0.81	0.75	0.74	1	0.76	0.72	0.76
R_1_ (Ω cm^2^)	0.53	80.1	79.1	85	0.63	70.5	80	75.5
C_1_ (F cm^−2^)	-	1.89 × 10^−6^	3.54 × 10^−6^	7.61 × 10^−7^	-	2.12 × 10^−6^	1.4 × 10^−6^	7.41 × 10^−7^
Q_2_-Y_o_ (Ω s^n^ cm^−2^)	0.1	2.34 × 10^−5^	6.3 × 10^−5^	4.9 × 10^−6^	0.1	3.25 × 10^−5^	6.3 × 10^−6^	5.1 × 10^−6^
Q_2_-n	0.8	0.82	0.86	0.83	0.77	0.80	0.85	0.85
R_2_ (Ω cm^2^)	3.5	5.8 × 10^5^	1.2 × 10^6^	1.4 × 10^6^	0.66	1.17 × 10^5^	1.8 × 10^5^	2.1 × 10^5^
C_2_ (F cm^−2^)	-	5.12 × 10^−5^	7.6 × 10^−5^	7.16 × 10^−6^	-	4.53 × 10^−5^	1.1 × 10^−5^	5.5 × 10^−6^
δ (nm)		136	800	980	-	154	1100	1270

**Table 4:** The values of prepared groups of flat band potential E_FB_ and donor density N_D_.

Samples	E_FB_ (V_SCE_)	N_D_ (cm^−3^)
Y_1_ (HCl)	0.47	1.66 × 10^19^
Y_2_ (HCl)	0.6	4.1 × 10^17^
Y_3_ (HCl)	0.6	4.0 × 10^17^
Y_1_ (H_2_SO_4_)	0.19	4.4 × 10^20^
Y_2_ (H_2_SO_4_)	0.5	1.01 × 10^18^
Y_3_ (H_2_SO_4_)	0.4	2.0 × 10^17^

## Data Availability

Data sharing is not applicable to this article.
